# Contemporary diagnosis of lower urinary tract dysfunction.

**DOI:** 10.12688/f1000research.16120.1

**Published:** 2019-05-09

**Authors:** Peter Rosier

**Affiliations:** 1 Department of Urology, University Medical Center Utrecht, Utrecht, The Netherlands

**Keywords:** Guidelines, diagnosis, urinary incontinence, lower urinary tract dysfunction, lower urinary tract symptoms, LUTS, OAB, BPH

## Abstract

**Introduction: **Diagnosis of lower urinary tract (LUT) dysfunction starts with categorization in clinical syndromes, and initial management is based on the assumptions about pathophysiology that these syndromes contain. However, clinical practice guidelines are ambiguous in clinical specialists’ diagnosis of dysfunction after failure of initial management. This is a narrative and critical review of the existing evidence, and the aim is to suggest practice improvements in the process of clinical specialists’ diagnosis for patients resistant to initial management.

**Methods and Results: **Evidence is collated on the basis of the author’s personal preference in combination with good clinical practice general principles. Statements and suggestions to improve reflect personal opinion. For two groups of patients with LUT dysfunction, the strategy of initial diagnosis is summarized and desirable principles of secondary care diagnosis are discussed. More specifically, a structure for the contemporary care of women with signs and symptoms of urinary incontinence is described and for that of the group of men older than 45 years with symptoms of LUT dysfunction.

**Conclusions: **Urodynamic testing is the undisputed gold standard for objective assessment and is the only way to stage and grade the dysfunction. Clinical practice guidelines and clinical specialists are too modest about the use and applicability of objective or urodynamic testing for referred persons with LUT dysfunction that is resistant to initial pragmatic management. Objective assessment and diagnosis are mainstays in secondary care, and the indication to perform objective assessments in patients with LUT dysfunction should be advised much more specifically in guidelines and practice recommendations.

## Introduction

The normal lower urinary tract (LUT) stores urine and is able to evacuate this at suitable moments. It is likely that evolutionary advantage gave humankind this ability over continuously leaking urine or very frequent voiding. Leaving too many smell traces is not good in the wild. When born, and even earlier, a child has the abilities to store and void; however, the bladder itself dictates the frequency in early life. An autonomic (pontine-sacral) reflex is built in to ensure this ^1^. This autonomic reflex becomes controllable during potty training and social continence is the endpoint. Anatomy, physiology, or function (or a combination of these) may be or become abnormal and result in signs and symptoms of dysfunction. Dysfunction of the LUT has many faces and I discuss two important types in this article with the aim to give arguments to raise the standard of care with regard to diagnosis in secondary—referred—care. These two groups are very prevalent and the recommendations in the guidelines for secondary care management are especially ambiguous about the relevance of adequate objective diagnosis for these: adult women with symptoms of urinary incontinence (UI) or too frequent voiding (or both) and adult men older than 45 years of age with symptoms of LUT dysfunction. I present a narrative, personal, and critical review of the evidence and of general contemporary good medical principles regarding the diagnostic process for patients with LUT dysfunction and the ultimate aims are to improve care and outcome and to reduce (potential) harm for these patients.

Persons with LUT dysfunction may seek care for their symptoms. Undoubtedly, there is an individual threshold for the decision to seek professional care ^2^ and without a doubt this threshold has wide intra-individual variation. Any person with symptoms who arrives at professional care enters a form of diagnostic process. Diagnosis of LUT dysfunction would first of all try to separate whether the dysfunction is secondary: related to the person’s medication (given for other diseases), to other diseases (for example, neurological, urinary tract inflammation or neoplasm), or to other dysfunction (for example, immobility or mental incapability to go to the toilet). Subsequently, the signs and symptoms that the patients report are categorized, so that a useable differential diagnosis can be obtained. I discuss women with symptoms and afterward the men with symptoms of LUT dysfunction.

## Women with symptoms of urinary incontinence

The initial workup of a woman who presents with UI includes history and clinical exam to be completed with a drinking voiding diary, reporting at least 24 hours, as is recommended in every practice guideline. The information gathered is useful to get a first impression of the likelihood of the dysfunction that is responsible for the symptoms ^3^. UI by itself is not life-threatening and when this sign of dysfunction is (with reasonable certainty) not the result of other diseases, it is possible to start an initial management, usually directed either to the pelvic floor—striated—muscles or to the bladder muscle (m. detrusor) ^4^.

Initial conservative management is usually life style or fluid intake management (or both) with the help of the voiding diary. Further conservative management is based on, for example, pelvic floor muscle training or medication (or both) to alleviate detrusor overactivity. When the patient reports that this is not successful, a referral to clinical specialist care may follow sooner or later in a proportion of patients. Transition from primary care to secondary care means, in common good medical practice, that objective confirmation of disease or dysfunction is sought, potentially (but not rarely) leading to a revision of the earlier diagnosis, which has been based on clinical signs and symptoms only.

However, the contemporary guidelines meant to support medical specialist care are surprisingly ambiguous regarding specific diagnosis of dysfunction for women with UI or too frequent micturitions or both ^5– 7^. Laboratory tests, imaging, and function testing are mainstays of the diagnostic process in medical specialist care in hospitals where radiologists, clinical chemists, bacteriologists, and so on are renowned specialists. Laboratory tests are relevant for women with UI to exclude hematuria or renal dysfunction (or both), and sometimes imaging of the kidneys is relevant. UI may be the result of detrusor muscle overactivity (detrusor overactivity incontinence) or of weakness of the bladder outlet closure mechanism (stress UI) or both. However, imaging of the LUT or the pelvic muscle closure mechanism (or both) with lateral cystography ^8^, which is abandoned nowadays, but also with magnetic resonance imaging ^9^ or perineal ultrasound ^10^ has never given results that were specific or sensitive enough to predict the type of UI dysfunction. Yes, the studies referred to here have been able to show elements of pathophysiology of—especially—stress UI; however, none of these has shown any relevance for the exclusion of the detrusor (overactivity) causing the UI. And none of these has been shown to be helpful in the exclusion of abnormalities of bladder-filling sensation or of voiding problems. Furthermore, not much progress has been made in subtyping or staging and grading of the continence dysfunction on the basis of imaging.

Staging and grading of the dysfunction are relevant for the selection of treatment in almost every area in health care or disease. Little is known about the staging or grading of UI. The amount of urinary leakage can be assessed in a standardized way ^11^ but this is to be regarded as a clinical staging. Apart from the fact that the amount of urinary leakage, as measured with a standard pad weighing test, associates only weakly with bother ^12^, this test is imperfect in the assessment of the type of dysfunction, as dichotomized above ^13^. Bother is another element of clinical staging. However, some women may have, for example, a very little amount of urine loss and experience a high degree of bother. Every clinician is well aware of the lack of association of expressed symptoms and existing pathology and knows that very serious (for example, malignant) disease can exist without any symptoms and vice versa.

It is difficult to define, on the basis of clinical staging, whether incontinence is severe or not severe. Objective criteria to separate severe from not severe UI do not exist at present. If severe would be translated as being more difficult to treat or as requiring a specific treatment, very low leak-point pressure (or intrinsic sphincter deficiency) would potentially lead to a specific management ^14– 16^. However, it is never scientifically demonstrated that, for example, larger-volume UI is more difficult to treat than small-volume low-frequency urine loss.

Urodynamic testing results in objective information about the function of the LUT. Not only continence function but also detrusor volume adaptation (compliance), bladder-filling sensation, and leak-point pressures can be determined during the filling phase. Furthermore, detrusor contraction strength and bladder outflow resistance during voiding become quantifiable ^17^.

Very low leak-point pressure would, as mentioned above, be a relevant and specific outcome of urodynamic testing. However, UI is not always observable or provocable during urodynamic testing but I do not regard this as a problem. UI is a symptom that is hardly ever presented as a false positive and it is reproducible without any equipment. The most important goal of urodynamic testing in patients with clinical stress UI (syndrome) is to try to exclude all LUT dysfunctions, except stress UI ^18^. On the other hand, the practice of provocation of (stress) UI during a clinical exam has been described in very many ways. During urodynamics, stress provocation is not better standardized. Only recently has there been an initiative to “uniformize” the clinical cough stress test ^19^.

Urodynamic diagnoses as detrusor overactivity, lack of filling compliance, lack of sensation, or ineffective voiding, would lead to the diagnosis of “not typical” stress UI and would have relevance when the patient has, for example, too frequent micturitions as (one of the) symptom(s) ^18^. Not typical stress UI should be treated specifically, directed to the predominant pathophysiology and individually based, on objective assessment.

Direct measurements of bladder outlet pressure (urethral pressure) in diverse ways (for example, profile) have demonstrated plausible results; the lower the “sphincter” pressure, the lesser the likelihood of continence. However, the diagnostic specificity is, like imaging, too low to discriminate between two types of UI and the remainder of the LUT function remains obscure. Patients with urodynamically confirmed genuine stress UI have been considered the ideal or only candidates for surgery since a 1980 publication by Cardozo and Stanton ^18^, especially in an era when surgery was invasive and required lower abdominal incision. The new century began, 20 years later, with the introduction of the tension-free—mid-urethral—vaginal tape for the treatment of “genuine stress urinary incontinence” ^20^. This treatment was introduced as a day-case procedure with local anesthesia, and outcomes were similar to those of the gold standard -Burch operation. This caused a revolution in the treatment of female stress UI but also lowered the threshold to surgically intervene. The diagnosis of genuine -or urodynamic stress UI, based on urodynamic exclusion of other or coexisting LUT dysfunctions, has gradually been slimmed down to “stress UI”, leaving out “genuine” and becoming a —yet poorly defined—clinical syndrome on the basis of subjective signs and symptoms ^21^. As a counterpart, the symptoms are frequently expressed with the existence of another common dysfunction, in combination with symptoms of UI; urodynamic detrusor overactivity were captured in a syndrome as well; the overactive bladder syndrome officially introduced as a syndrome ^22^, however alike “stress incontinence” applied in a simplified way by many, confusing “a syndrome” with “a disease”. Although patients with detrusor overactivity frequently report symptoms of this syndrome, the syndrome is not at all specific for detrusor overactivity. Patients are—expectably—not able to precisely describe their dysfunction very well ^23^ and their descriptions do not automatically fit the medical concepts. Consequently, a “mixed incontinence” category, based on reported symptoms, arrived without any pathophysiological foundation and no evidence of any specific management ^24^.

Clinical syndromes are relevant, needed, and helpful to ease communication among professionals and are helpful to initiate conservative management. However, with regard to UI syndromes, there is a wealth of evidence to confirm that these are not specific and not sensitive enough to delineate the pathophysiology, especially not in the referred population ^25^. Failure of initial conservative management in primary health care would first be considered a failure of diagnosis (as a result of ex-juvantibus management) and leads to reconsideration of the diagnosis or referral to secondary care (or both). In general, referral implies assessment of objective evidence for disease or dysfunction by making use of the abovementioned supporting specialists. This is hardly the case for patients with UI where syndromes have become the mainstay of diagnosis. Moreover, a good definition of failure of initial management is lacking ^26^ and systematic follow-up diagnosis is deemed to require further phenotyping ^27^. That a specific urodynamic diagnosis is helpful and needed in patients with failure after initial management is demonstrated in a prospectively randomized study in which 36% of the patients included were shown to have urodynamic results that (outside the construct of this trial) would not lead to the one of the interventions tested. More precisely, only 64% of the patients (231 out of 364) included with the overactive bladder syndrome showed detrusor overactivity on urodynamic testing and eventually would be potential candidates for botulin toxin injections or sacral neuromodulation. The patients without detrusor overactivity failed on initial management for the overactive bladder syndrome, and the urodynamic testing has demonstrated why. This is in agreement with all of the evidence that demonstrates that the syndromes are only weak indicators of pathophysiological diagnosis ^25^.

Clinical staging on the basis of signs and symptoms is relevant on the one hand; on the other hand, staging and grading of pathophysiology are equally important in secondary care to be able to individualize management, to prevent unnecessary surgery and interventions, and to improve management outcomes for all patients after failed initial management. Objective assessment of (LUT) dysfunction (diagnosis) is not designed to “improve the outcome of surgery”; it is designed to allocate individuals to a management strategy with a demonstrated mechanism of action toward their dysfunction.

Contemporary diagnosis of UI in adult women is recommended to be based on signs and symptoms, reported or observed (or both) ^5– 7^, but it seems worthwhile to better delineate the syndromes and better define failure of initial management ^21^. Clinical specialist diagnosis of patients with failed initial treatment is endangered to fall prey to nihilism. Too many patients are advised a ‘one size fits all’ invasive management without objective and individualized staging and grading of the dysfunction. Staging and grading of dysfunction and disease are mainstays in secondary care, and the patients with failure after initial management for their UI deserve objective assessment because they worry about the origin of their symptoms ^23^ and expect information about cause and about the specific and individualized management of their dysfunction ^28^.

## Men with lower urinary tract dysfunction

Elderly men with symptoms of LUT dysfunction have “prostatism”. This simple concept was apparently valid until almost 1980. The prostate tends to continue growing after the fourth decade of a man’s life and as a result of human anatomy the growing prostate may cause urinary tract symptoms ^29^. That this epidemiological coincidence of later-life prostate growth and “prostatism” as the syndrome has led to unjustified causation and generalization was shown in a pivotal article ^30^. Not all LUT symptoms are the result of bladder outflow obstruction (BOO) in every man. Not all men have BOO, and elderly men may have LUT symptoms without prostatic enlargement. Surgery for prostatism was gradually replaced with less invasive and medical management of LUT dysfunction. Urodynamic assessment (confirmation) with grading of BOO has been used to validate these new treatment options designed specifically to relieve BOO ^31– 34^. Apart from urodynamic outcomes, test-retest evaluations of urodynamic quantification of BOO have been published ^35, 36^. It was also confirmed that prostate size is associated with the likelihood of BOO ^37^. Flow rate is reduced in many men with symptoms of LUT dysfunction, and the combination of prostate enlargement and reduced flow rate with or without post-void residual is a better predictor of BOO than reported symptoms alone ^38– 40^. Grading of BOO with a pressure flow plot has become standard for elderly males. ^41^, In a simplified form of pressure flow quantification, the International Continence Society (ICS) provisional bladder outlet index (BOOI or initially: A-G number), introduced herein can be used to numerically quantify the grade of BOO. Furthermore, as outlined above, grading of the effect of management directed to the outflow obstructive component of the dysfunction as well as (safety) monitoring of expectative management is possible. Pressure flow analysis is clearly relevant in the management of elderly male patients and would lead patients (and physicians) away from treatment on the basis of symptoms and bother ^42^. Sometimes, reports give a wide variation in prostate sizes and flow rates that cannot very likely associate with BOO at the lower end of the prostate sizes and at the upper end of the flow rate ^43^. In many published reports, urodynamics has not clearly been used to select men for invasive management or to exclude them if no BOO was diagnosed. This leaves the possibility that a patient was operated with a technique aiming at reduction of outflow resistance without relevant BOO present.

For referred elderly men with LUT dysfunction persisting with or after initial management, diagnosis should not only confirm or exclude BOO. Urodynamic testing also gives the possibility to quantify detrusor voiding contraction strength as well as detrusor volume adaptation and relaxation. Detrusor overactivity is very prevalent in elderly men and is undeniably intermingling in the male LUT dysfunction syndrome. Elderly men without (high-grade) BOO and with detrusor overactivity would profit from specific medical management. Men with high-grade BOO and detrusor overactivity may deserve a less expectative management than men with a moderate grade of BOO without detrusor overactivity. Regrettably, this stratification of disease, as was already introduced in 1979 ^30^, has been overwhelmed with the (enthusiastic) introduction of minimally invasive and medical management options for elderly men with symptoms but also for these options: “one size fits all” –management strategies do not exist in health care, especially not when initiated on the basis of symptoms only. Physiological demonstration of the mechanism of action and of effect size should be the basis of the introduction of every new management option (surgical, instrumental, or medical) for LUT dysfunction. Individualization of management on the basis of objectively assessed pathophysiology should be the gold standard in every clinical specialist’s practice.

## Conclusions

Contemporary diagnosis of LUT dysfunction, especially of UI or too frequent voiding in women over 40 years of age (or both), and of symptoms of dysfunction in men over 45 years of age begins with a LUT syndrome diagnosis on the basis of clinical epidemiology and reported symptoms and signs, including fluid balance. Initial non-invasive management can safely be based on this. However, I plead that, if initial management fails, both categories of patients deserve objective assessment of their LUT function as the basis for further specific and individualized management. Contemporary medical specialists’ guidelines are far too modest about the indication and applicability of objective testing for persons with LUT dysfunction, and existing evidence should be included. Objective testing is inescapable and invaluable in secondary health care on the basis of good practice, and urodynamic testing is the undisputed gold standard for this objective assessment and the only way to stage and grade the dysfunction. Individualized management on the basis of objective diagnosis is the paradigm of modern health care. Functional urology should not lag behind.
